# Transfusion-related acute lung injury

**DOI:** 10.4103/0973-6247.67019

**Published:** 2010-07

**Authors:** Ramakant Dixit, Sidharth Sharma, A. R. Parmez

**Affiliations:** Department of Respiratory Medicine and Tuberculosis, J. L. N. Medical College, Ajmer, India

Sir,

Transfusion-related acute lung injury (TRALI) is an uncommon, probably underrecognized complication of transfusion of plasma-containing blood components, which is characterized by acute respiratory distress. It is a clinical diagnosis and may be diagnosed when acute lung injury (ALI) occurs during or within six hours of transfusion in a patient without pre-existing ALI, in the absence of temporally related alternative risk factors for ALI (e.g., sepsis, shock, cardiac failure, etc.).[1] This communication discusses a case of TRALI in a middle-aged woman.

A 52-year-old female patient with a diagnosis of inoperable carcinoma of the uterine cervix, received whole blood transfusion, in view of severe anemia. Before blood transfusion, she was alert, oriented, with no cardiorespiratory abnormality on clinicoradiological evaluation. Her vital parameters were normal and total leukocyte count was 8700/mm^3^ with normal blood biochemistry. After receiving approximately 200 ml of blood for over two hours, she complained of breathlessness, which progressively increased over the next 20 minutes. On examination she was febrile, tachypneic (respiratory rate 32/minute), slightly cyanotic, with a pulse rate of 120/minute, blood pressure 90/60 mmHg, and SpO_2_ 68%. The jugular venous pressure (JVP) was normal and chest auscultation revealed bilateral basal fine inspiratory crackles with normal breath sounds. Sinus tachycardia was noted on an electrocardiogram (ECG). An urgent X-ray chest showed bilateral patchy infiltrates in the mid- and lower zones with normal heart size, suggestive of noncardiogenic pulmonary edema. Echocardiography also ruled out cardiogenic dysfunction. Hematological investigations at this stage showed a total leukocyte count of 3200/mm^3^, with no evidence of any hemolytic process. In the absence of other mechanisms to explain ALI, a diagnosis of TRALI was made.

The blood transfusion was stopped immediately and she was managed with oxygen therapy and intravenous fluids that resulted in a gradual improvement of dyspnea and oxygen saturation. She became completely chest asymptomatic after three days, with a normal chest X-ray.

The reported incidence of TRALI in medical literature is one in 5000 transfusions and this may actually be much higher as TRALI is usually an underrecognized and underreported entity.[2] TRALI has been associated with antibodies to WBCs in transfused blood components. Less frequently, it has been associated with leukocyte antibodies in the transfusion recipient. It has also been linked to the infusion of biologically-active lipids in stored cellular blood components.[3]

A mild form of TRALI can present with dyspnea and fever, while more severe forms can have severe respiratory distress that can quickly progress to respiratory failure. Other features include hypotension, frothy endotracheal secretions, and so on. Transient leucopenia, as noted in our patient, is occasionally observed and is believed to be due to the sequestration of leukocytes in pulmonary circulation. It is important to distinguish TRALI from volume overload, as the treatment of the two conditions is markedly different. Diuretics are contraindicated in TRALI and there is no role for steroids also. Severe forms of TRALI may require mechanical ventilation.[3]

Suspected cases of TRALI should be reported to the blood bank, to permit evaluation and deferral of high-risk donors, especially multiparous females. TRALI can be best prevented by avoiding unnecessary blood-product transfusions in selected populations.
